# The role of immunomodulators in treatment-resistant depression: case studies

**DOI:** 10.1038/s41420-022-01147-6

**Published:** 2022-08-17

**Authors:** Charles W. Beckett, Maria Victoria Niklison-Chirou

**Affiliations:** 1 Department of Pharmacy and Pharmacology, University of Bath, Bath, BA2 7AY UK; 2 Centre for Therapeutic Innovation, Department of Pharmacy and Pharmacology, University of Bath, Bath, BA2 7AY UK

**Keywords:** Target identification, Pharmacodynamics, Chronic inflammation, Neuroimmunology, Depression

## Abstract

Depression is a common mental disorder affecting more than 264 million people worldwide. The first-line treatment for most cases of depression are selective serotonin reuptake inhibitors (SSRIs), such as sertraline, reboxetine and fluoxetine. Recently, it has been found that one-quarter of depressed patients have excessive activation of the immune system. This potentially warrants sub-categorisation of depressed patients into inflammatory and non-inflammatory subtypes. Such a sub-category of depression already exists for those not responding to various traditional antidepressants and is known as treatment-resistant depression. Those with treatment-resistant depression are far more likely to have raised inflammatory markers relative to those whose depression is treatment-responsive. Chronic, low-level inflammation seems to trigger depression via a multitude of mechanisms. These include kynurenine pathway and microglial cell activation, resulting in a reduction in hippocampal volume. Raised inflammatory cytokines also cause perturbations in monoaminergic signalling, which perhaps explains the preponderance of treatment resistance in those patients with inflammatory depression. Therefore, if treatment-resistant depression and inflammatory depression are semi-synonymous then it should follow that anti-inflammatory drugs will display high efficacy in both sub-types. Ketamine is a drug recently approved for use in depression in the USA and displays a particularly good response rate in those patients with treatment resistance. It has been suggested that the antidepressant efficacy of ketamine results from its anti-inflammatory effects. Ketamine seems to produce anti-inflammatory effects via polarisation of monocytes to M2 macrophages. Furthermore, another anti-inflammatory drug with potential use in treatment-resistant depression is Celecoxib. Celecoxib is a long-acting, selective COX-2 inhibitor. Early clinical trials show that Celecoxib has an adjuvant effect with traditional antidepressants in treatment-resistant patients. This paper highlights the importance of classifying depressed patients into inflammatory and non-inflammatory subtypes; and how this may lead to the development of more targeted treatments for treatment-resistant depression.

## Introduction

Depression is a psychiatric disorder that affects mood, behaviour and overall health. The treatment for depression is a combination of counselling and pharmacotherapy. Depression treatment can be challenging due to drug side effects. Importantly, there is a group of depressed patients who do not respond adequately to multiple courses of appropriate pharmacotherapy, patients with treatment-resistant depression. Lack of response to antidepressants increases the risk of suicide, prolongs unnecessary suffering and is a large healthcare burden [1]. This makes finding alternative treatments for treatment-resistant patients a healthcare imperative. In this perspective, we will discuss the latest discoveries in treatment-resistant depression and how immunomodulatory drugs could be used to improve treatment responses.

## Why would immunomodulators be useful in depression?

A comprehensive literature shows that depression is linked to an activation of the immune system. The primary evidence for this comes from measures of immune cytokines. Cytokines associated with Th1 activation, including tumour necrosis factor (TNF)-α, interleukin (IL)-1β and IL-6, are raised in the cerebrospinal fluid and plasma of depressed patients. Furthermore, activated microglial cells are found in patients during a depressive episode, relative to healthy control [2], and other biomarkers of inflammatory states, such as low serum iron and raised body temperature, are seen in major depression [3].

Worthy of inquiry is in which direction the relationship between inflammation and depression is causal. It is commonly known that depression-like behaviour can be induced in animals by the injection of lipopolysaccharide (LPS), which is highly pro-inflammatory [4]. In addition to this, clinical use of interferons in multiple sclerosis, among other disorders, can cause depression as a side effect. This implies that the inflammatory response can be causal in depression [5].

Further evidence for an immune system-depression link is provided by genomics data. Many genetic polymorphisms have been associated with depression but the existence of genetic roots for depression is somewhat perplexing. Through evolutionary history, depression would have increased one’s risk of death from suicide [6, 7]. This begs the question, therefore, as to why these genes would continue to persist in the gene pool? One answer is that depression-associated genes could provide a dual function. Single nucleotide polymorphisms identified by candidate genes and genome-wide association studies to be correlated with depression risk are regularly found in immune-related genes. Indeed, 8 of the 10 gene variants that most strongly increase depression risk also have immune or inflammatory function [3]. Such evolutionary trade-offs are common, as is the case with the malaria-protective effects of the haemoglobin mutation that causes sickle cell disease for example [8]. The fact that inflammation appears to trigger depression is likely more than just an evolutionary trade-off, however, as it has been proposed that depression is a prolonged form of sickness behaviour. Indeed, the symptoms of major depression are remarkably akin to sickness behaviour. Both are characterised by sleep pattern change (insomnia, hypersomnia), appetite changes, reduced sociability (social withdrawal), irritability, low mood and reduced motivation and interest in daily activities (anhedonia) [3].

The primary mechanism proposed to mediate Th1 immune response-triggered depression is the activation of indoleamine 2,3-dioxygenase (IDO). IDO is an enzyme involved in tryptophan metabolism and is up-regulated and activated by Th1-associated cytokines, particularly IFNγ [9, 10]. It generates kynurenine from tryptophan, diverting tryptophan away from serotonin synthesis. Kynurenine and its downstream metabolites are neurotoxic. This is due to the direct agonism of *N*-methyl-d-aspartate (NMDA) receptors and activation of glutamate and reactive oxygen species release from microglial cells. The resultant excitotoxicity and oxidative stress cause tissue damage, particularly in the hippocampus. Reduced hippocampal volume is a well-characterised marker seen in the brains of depressed people [11–14]. Further support for this hypothesis is found in the fact that LPS was unable to engender depression-like behaviour in IDO1 knockout mice, suggesting inflammation-induced depression is IDO-dependent [15].

In addition to activation of the kynurenine pathway causing hippocampal neurotoxicity, inflammation also seems to attenuate hippocampal neurogenesis. IL-6, IL-1 and TNFα, for example, all suppress the neurotrophin, brain-derived neurotrophic factor (BDNF). BDNF is known to ameliorate depressive symptoms, in part by increasing hippocampal neurogenesis, which is consistently decreased in depressed individuals [16]. Also, TNF-α increases the activity of nuclear factor kappa B, which further suppresses neurogenesis [17]. Furthermore, the inflammation affects monoaminergic signalling in the brain via mechanisms beyond those associated with IDO-induced tryptophan depletion. IL-1 and TNF-α increase phosphorylation of the serotonin transporter (SERT). This leads to increased translocation of SERT into the neuronal membrane, resulting in increased serotonin reuptake and reduced response to SERT-blocking antidepressants [18]. Furthermore, chronic, low-grade inflammation seems to reduce dopamine synthesis, packaging and release. Reduced dopamine neurotransmission increases depression risk, particularly symptoms of anhedonia, fatigue and psychomotor retardation [19].

Despite the link between inflammation and depression being incontrovertible, there is a large degree of heterogeneity between patients regarding inflammatory status. Around one-quarter of patients with depression have raised low-level inflammation [20]. This suggest that depressed patient can be categorised into non-inflammatory and inflammatory subtypes [21]. Furthermore, akin to the heterogeneity observed regarding the presence of inflammation in depressed populations, traditional antidepressants display a large degree of heterogeneity in their efficacy. On average, traditional antidepressants seem to show around a 25% remission rate, 75% response rate and 25% non-response rate, though this differs substantially from study to study [1, 22, 23]. Interestingly, treatment-resistant depression is far more often accompanied by increased Th1 cytokines relative to that which is treatment-responsive [24, 25]. This data suggests that the inflammatory depression subtype is also characterised by resistance to traditional antidepressants and, therefore, investigation of the efficacy of immunomodulators in treatment-resistant depression would be prudent.

**Fig. 1:**
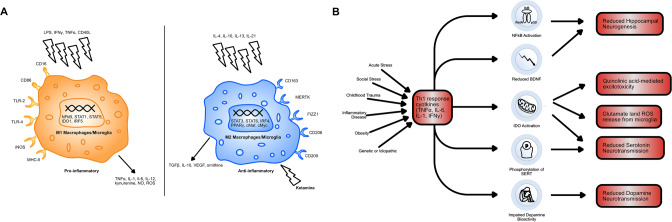
Th1 dominance in depression and its amelioration by ketamine. **A** A diagram comparing M1 and M2 type macrophages. M1 macrophages are activated by pro-inflammatory signals, including LPS, Th1 cytokines (IFNγ, TNFα) and Th1 cell surface proteins (CD40L). M2 macrophages are activated by Th2 cytokines (IL-4, IL-10, IL-13, IL-21) and by ketamine. M1 macrophages produce pro-inflammatory cytokines, as well as nitric oxide (NO) and neurotoxic kynurenine and reactive oxygen species and thus mediate the Th1 response. Ketamine causes the polarisation of monocytes to M2 macrophages. This acts to promote the release of anti-inflammatory TGFβ, IL-10 and ornithine. It also reduces monocyte differentiation to M1 macrophages, hampering the effects of Th1 dominance. **B** A schematic showing the possible causes of Th1 dominance in patients with treatment-resistant depression and the downstream consequences of this excessive Th1 immune activation. Indoleamine 2,3-dioxygenase (IDO) activation is central to inflammation-induced depression via its effects on tryptophan metabolism and generation of neurotoxicity. IDO-mediated changes in tryptophan metabolism reduce serotonin, as does phosphorylation of serotonin transporters (SERT). Brain-derived neurotrophic factor (BDNF) exacerbates the negative effects of IDO activation on hippocampal volume by reducing hippocampal neurogenesis, as does nuclear factor kappa B (NFkB) induction.

The salience of an inflammatory depression subtype, appropriately targeted by immunosuppressives, is further supported by a number of other noteworthy facts from the literature. Firstly, depression that is co-morbid with previous childhood ill-treatment is associated with an increase in inflammation and is more often treatment-resistant [26]. That is, relative to depressed patients with a more normative parental environment. Similarly, acute stress seems to increase immune function, in direct contrast to chronic stress [27]. This is important in social stress, which increases pro-inflammatory cytokines in both humans and rodents [28, 29] (Fig. 1B). Furthermore, trials of cytokine inhibitor use in depression, such as infliximab (anti-TNFα) and sirukumab (anti-IL-6), selectively display efficacy in patients with low- inflammation levels prior to treatment [30, 31]. This selective efficacy will be a theme when discussing other immunomodulators (ketamine and celecoxib) during the rest of the paper.

### Ketamine

Ketamine is a drug that has been used for many years as a fast-acting non-barbiturate general anaesthetic. It also has well-established use in hypotensive shock, reactive airway disease, analgesia and procedural sedation. It is primarily an NMDA receptor antagonist, but its pharmacodynamics are complex. It also shows interactions with opioid, cholinergic, purinergic and adrenergic receptors, as well as with ion channels not gated by endogenous ligands [32]. More recently, ketamine has found its purpose in depression, being approved by the US food and drug administration in 2019 [33].

Ketamine has a remarkably high response rate in treatment-resistant depression (~65%) but the exact mechanisms underlying its antidepressant effects are unknown, particularly as other NMDA receptor antagonists possess non-comparable effects [34, 35]. While a multiplicity of mechanisms have been proposed, such as effects on opioid and AMPA receptors [36–38], ketamine seems to produce a number of its effects via immunomodulatory mechanisms. A systematic review of 9 human studies and 22 animal studies [39] found that ketamine consistently produced reductions in IL-1β, IL-6 and TNFα. Furthermore, in all but one study in which it was measured ketamine was found to decrease IDO activity and reduce the prevalence of downstream neurotoxic metabolites [39]. Moreover, the magnitude of reduction in IL-6 and IL-1β is associated with the magnitude of ketamine’s antidepressant effect [40].

Little is known about ketamine’s immunomodulatory mechanism. It is likely to be the result of some direct effect on leucocytes [41]. This is evidenced by the fact that ketamine can reduce pro-inflammatory cytokine production in isolated human blood. One current hypothesis as to how ketamine does this, is that it causes macrophage polarisation to an M2 phenotype [42]. Macrophages can be categorised into M1 and M2 type macrophages. Th1 cells induce differentiation to M1 macrophages via the expression of IFN-γ and CD40 ligand. These macrophages are highly pro-inflammatory. Meanwhile, Th2 cells induced differentiation to anti-inflammatory M2 macrophages via the expression of IL-4 and IL-13. M1 macrophages preferentially produce many of the pro-inflammatory immune cytokines seen in depression, such as IL-6 and TNF-α, as well as pro-inflammatory nitric oxide (NO) and reactive oxygen species (Fig. 1A). Therefore, by ketamine polarising macrophages to the M2 type, it may be opposing the downstream effects of the Th1 dominance observed in depression [43, 44]. Furthermore, IDO is more highly expressed in M1 macrophages, which may explain how ketamine reduces its activity [45]. The exact mechanism via which ketamine causes monocytes to differentiate into M2 macrophages is unclear but it seems to occur via an mTOR-dependent mechanism and involved increased expression of CD163 and MERTK [42].

Microglia also possess an M1 and M2 bifurcation in phenotype and LPS induces differentiation of microglia to an M1 type via activation of toll-like receptors, hence its neuroinflammatory effect [46]. Ketamine blocks LPS-induced M1 differentiation in both peripheral and central nervous system macrophages [47, 48]. Furthermore, M2 microglia preferentially produce transforming growth factor (TGF)-1β, which produces anti-inflammatory effects in the brain. Ketamine can prevent reductions in TGF-1β levels induced by chronic social defeat stress in mice (Fig. 1B). Meanwhile, the use of an anti-TGF-1β antibody in mice experiencing chronic social defeat stress blocks the antidepressant effects of ketamine [49].

### Celecoxib

Celecoxib is a long-acting, selective cyclooxygenase-2 inhibitor. It is of similar potency to ibuprofen but is used in patients with mild to moderate pain and/or with arthritis, who cannot tolerate the gastrointestinal side effects of traditional non-steroidal anti-inflammatory drugs (NSAIDs). Celecoxib works by inhibiting pro-inflammatory protein synthesis [50]. Downstream effects of this protein synthesis inhibition are facilitated by alterations in cell-cell interactions, vascular tone and permeability, cytokine production and receptor expression and leucocyte maturation, migration and survival [51].

Several studies had shown that Celecoxib is useful in treatment-resistant and inflammatory depression. Greater over-the-counter NSAID use is associated with reduced depression rates in the Danish population, suggesting NSAIDs may be antidepressant per se [52]. Furthermore, depression is associated with raised body temperature, perhaps suggesting a role of prostaglandin E2 in its pathophysiology [43, 53]. The effects of NSAIDs on immune cytokines also indicate potential efficacy. As mentioned previously, IL-6 is the most commonly raised immune cytokine in depression. The synthesis of IL-6, downstream of CD40 activation in B cells, is dependent upon COX-2 [54]. This, among other mechanisms, means that NSAIDs reliably reduce levels of IL-1 and IL-6, leading to a reduction in the Th1 response [55, 56]. Lastly, one of the ten gene polymorphisms most strongly associated with depression is found is *adcy3*, which codes for adenylate cyclase 3. Adenylate cyclase 3 plays an integral role in the signal transduction downstream of prostaglandin receptors, further providing evidence of a role for COX-2 inhibition in depression [3].

**Fig. 2:**
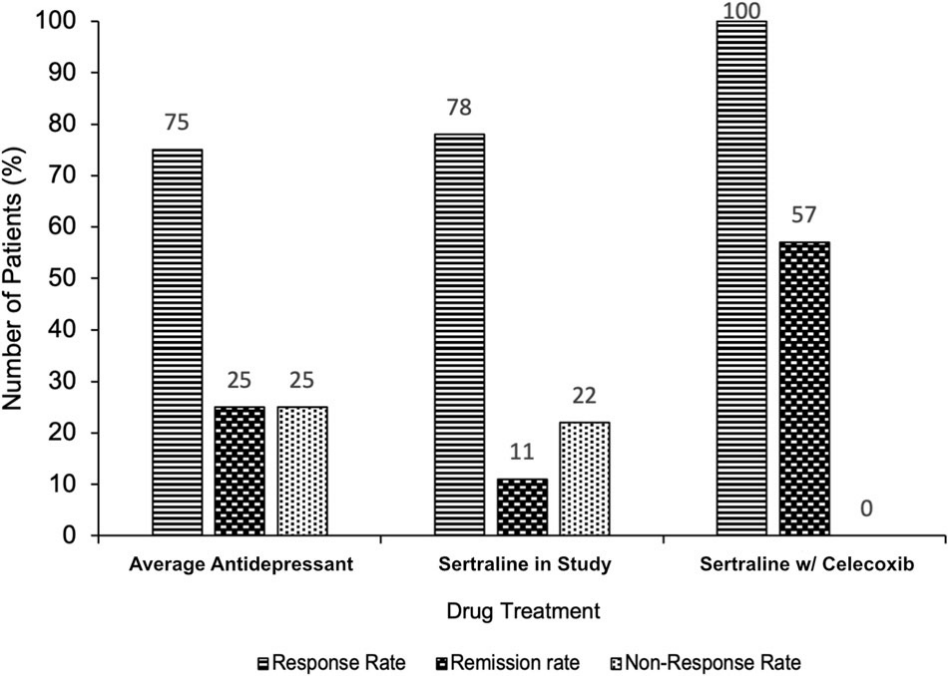
Approximate mean antidepressant responses in the literature versus responses to sertraline alone and sertraline with celecoxib observed by Majd et al., 2015. A bar chart showing the percentage response rate, remission rate and non-response rate to antidepressant treatment. Responses to three drug treatments are compared: the average first-line selective serotonin reuptake inhibitor (SSRI) (numbers derived from analysis of the wider literature [1, 22, 23], shown on the left, sertraline in drug-naive women (data taken from [58]), shown in the centre, and sertraline in combination with celecoxib in drug-naive women [58], shown on the right. The addition of celecoxib to SSRI treatment dramatically increases the percentage rate of response (100% taking celecoxib vs 75–78% taking placebo/nothing) and rate of remission (57% taking celecoxib vs 11–25% taking placebo/nothing). This provides evidence as to the efficacy of celecoxib as an adjunctive treatment and suggests it may actively target the treatment-resistant depression subtype.

Multiple clinical trials have been conducted using celecoxib in depressed individuals. In 2014, a meta-analysis of celecoxib trials was conducted [57]. Monotherapy and therapy adjunctive to traditional antidepressants in depressed individuals with and without co-morbid inflammatory disease were found to be superior to placebo and to traditional antidepressants alone [57]. It is worth noting that it is difficult to draw conclusions from trials in patients that had a co-morbid inflammatory disease (6 out of 11 of those included in the meta-analysis). This is because there is no mechanism to control for the potential that the antidepressant effect resulted indirectly via amelioration of discomfort (swelling, pain, etc.) rather than via some direct action. Saying this, more trials have been conducted since 2014 with some promising results. The effect of sertraline, a common drug to treat depression, with celecoxib versus sertraline alone in drug-naive depressed women was assessed [58]. Although no statistically significant difference in the mean reduction of Hamilton Depression Rating Scale scores was observed at eight weeks, the response rate and remission rate were much higher in the celecoxib group relative to the sertraline-only group. The response rate was 100% in the group taking both sertraline and celecoxib compared with 78% in the group taking sertraline alone. Furthermore, and perhaps most worthy of attention, the remission rate in the celecoxib group was 57% relative to 11% in the placebo group. This trial suggests that Celecoxib directly targets individuals who would normally be treatment-resistant (Fig. 2) Also, Celecoxib adjunct therapy was found to reduce serum IL-6 in depressed patients and the magnitude of the IL-6 reduction predicted the magnitude of the antidepressant effect [59]. Moreover, Celecoxib seems to target the IDO pathway. Higher kynurenine serum concentrations prior to treatment were found to predict remission in patients given Celecoxib adjunct therapy [60]. Lastly, Celecoxib monotherapy is better than placebo in rat models of depression and its use is associated with reduced levels of immune cytokines [61]. It also potentiates the effect of reboxetine and fluoxetine, common drugs to treat depression, on cortical noradrenaline and serotonin output in rats [62]. Considering this data, Celecoxib seems to be targeting treatment-resistant depression via the Th1-associated immune pathways.

Unfortunately, there are contradictory results within the literature. For example, the only long-term trial of Celecoxib monotherapy in depression conducted to date, found no treatment effect [57, 63]. This could be due to the lack of selection for individuals with inflammatory depression, rather than a lack of drug effect. On the other hand, the dearth of long-term proven efficacy and tolerability of Celecoxib in depression is concerning. While the absence of COX1 inhibition obviates the risk of gastrointestinal side effects to Celecoxib use [50], its selectivity could increase the risk of blood clotting due to preferential inhibition of prostacyclin vs thromboxane A2 synthesis [64]. Saying this, in the 2014 meta-analysis [57], the authors found no significant increase in cardiovascular incidence when using Celecoxib in depression, but the lack of long-term trials included in the study makes this finding unconvincing at this point. Also noteworthy is that while NSAID use is correlated with antidepressant effects, this is not a black and white finding. Chronic use of over-the-counter NSAIDs or the use of high doses of aspirin was associated with increased depression incidence [52]. While this is only a correlative finding, it could indicate that Celecoxib would not be applicable to long-term depression treatment.

## Conclusions and perspectives

Depression is a complex condition. It is well accepted that inflammation can be a core feature of depression. Treatment-resistant depression is a subtype of depression, frequently characterised by enhancement of the Th1 cell-mediated and inflammatory immune responses. This subtype of depression could be amenable to improvement with the use of immunomodulatory drugs. Th1 dominance seems to cause treatment-resistant depression by causing reductions in hippocampal volume, mediated by IDO activation, microglial activation and reduction in neurogenesis, and perturbations in monoaminergic neurotransmission. Clinical trials of immunomodulators in depression thus far have been thwarted by the lack of an inflammatory depression subtype classification but have still shown promising results nonetheless.

Ketamine is an exemplar of how effective a drug with immunomodulatory mechanisms can be in treatment-resistant depression. It seems to act via M2 polarisation of macrophages, which results in attenuation of the Th1 immune response. Celecoxib is a pharmacotherapy in earlier stages of development for treatment-resistant depression, which has shown exciting results. This is especially true when used as an ancillary to traditional antidepressants, notably increasing the response and remission rate. Further research is needed to elucidate the exact mechanism by which ketamine causes monocytes to differentiate into M2 macrophages and to what extent its efficacy in treatment-resistant depression is the result of immunomodulation. Moreover, future research should focus on confirming the efficacy, safety and tolerability of Celecoxib in long-term clinical trials in individuals with inflammatory depression.

## Data Availability

All the data used to support the arguments in this study are included within the article.
